# Targeting native and apo-sGC

**DOI:** 10.1186/2050-6511-14-S1-O17

**Published:** 2013-08-29

**Authors:** Mary Struthers, Subharekha Raghavan, Ronald Kim, Christopher Sinz, Sophie Roy, Michael Mendelsohn

**Affiliations:** 1 Merck Research Laboratories, Rahway, NJ 07065, USA

## 

Soluble guanylate cyclase (sGC), the receptor for nitric oxide (NO), is one component of the nitric oxide (NO)-sGC-cGMP signaling pathway, which plays a key role in the cardiovascular system regulating smooth muscle relaxation and vasodilation and has been implicated in remodeling events in the heart and vasculature. Triggered by the binding endothelial-derived NO to the prosthetic heme group, sGC in smooth muscle cells converts GTP to the secondary messenger cGMP. Small molecule activators of sGC have been identified that either have the ability to activate the native heme-containing form of the enzyme (Heme-Dependent activators) or have the ability to activate to heme-free or oxidized form of the enzyme (Heme-Independent activators). The therapeutic potential of activators of sGC is illustrated by the successful clinical evaluation by Bayer of a Heme-dependent activator riociguat in pulmonary arterial hypertension [1]. The characterization of both heme-dependent and heme-independent activators with regard to their biochemical interactions with the enzyme as well as preclinical in vivo activity in rodent models of systemic and pulmonary hypertension will be presented.
